# Safety and Effectiveness of a Crosslinked Hyaluronic Acid Filler in Korean Patients for the Correction of Nasolabial Folds: A Randomized, Patient- and Evaluator-Blinded, Paired Study

**DOI:** 10.1093/asj/sjag003

**Published:** 2026-01-08

**Authors:** Si-Hyung Lee, Eunsoo Park, Chong Hyun Won

## Abstract

**Background:**

Hyaluronic acid (HA) dermal fillers are widely used as a nonsurgical treatment for facial aging, including nasolabial folds (NLFs). Because demand for convenient aesthetic procedures increases, new HA products require rigorous comparison with established fillers.

**Objectives:**

The aim of this study was to assess the noninferiority and safety of a new crosslinked HA filler, SkinPlus-HYAL Implant Lidocaine (test), vs RESTYLANE Lidocaine (control) for temporary correction of moderate to severe NLFs.

**Methods:**

In this multicenter, randomized, patient- and evaluator-blinded, split-face study, 100 adults with moderate or severe NLFs received the test filler in 1 NLF and the control in the contralateral NLF. Efficacy was analyzed in the full analysis set (*n* = 93). The primary endpoint was the between-treatment difference in Wrinkle Severity Rating Scale (WSRS) scores at Week 24. Safety was evaluated in the safety set (*n* = 100). Group differences were analyzed using a 2-sample *t*-test.

**Results:**

At Week 24, mean WSRS scores were 1.85 ± 0.72 (test) and 1.84 ± 0.68 (control). The mean difference (test − control) was 0.01 ± 0.48, with an upper 97.5% 1-sided confidence limit of 0.2136, below the prespecified noninferiority margin of 0.29. Investigator-rated Global Aesthetic Improvement Scale (GAIS) scores at Week 24 and patient-rated GAIS scores at Week 8 favored the test filler. Local adverse events were more frequent with the test (92.00%) than the control (82.0%), but severe injection-site reactions were uncommon, and no serious adverse events occurred.

**Conclusions:**

SkinPlus-HYAL Implant Lidocaine was noninferior to RESTYLANE Lidocaine for correction of moderate to severe NLFs and maintained efficacy through 48 weeks. Despite a higher rate of local reactions, its overall safety profile was acceptable, supporting its use as an effective, safe option for facial aesthetic augmentation.

**Level of Evidence: 1 (Therapeutic):**

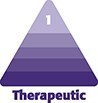

The global aesthetic market has seen continuous growth, driven by an aging population and increasing demand for minimally invasive anti-aging treatments. Among nonsurgical soft tissue augmentation procedures, hyaluronic acid (HA) dermal fillers remain a frequently utilized treatment because of their proven safety, biocompatibility, and reversibility.^1^

Nasolabial folds (NLFs) are typically caused by the loss of deep fat and subsequent loss of muscle contour in the midface, leading to sagging and the formation of wrinkles and folds.^2^ HA dermal injections successfully treat NLFs by providing volume to the targeted area and restoring the natural contour of the treated region.^3^ HA, a naturally occurring glycosaminoglycan, exerts its filling effect primarily through its high hydrophilicity and resulting volume expansion when injected into the dermal matrix.^4^ However, native HA is rapidly metabolized in vivo, necessitating chemical crosslinking to stabilize the gel structure, enhance resistance to enzymatic degradation, and prolong clinical efficacy.^5,6^ This process involves linking individual HA molecules to form a stable, durable gel network. The resulting highly crosslinked HA gel provides greater structural integrity and long-lasting results, which are key determinants of a filler's performance.^6^

The manufacturing parameters for HA fillers—including the degree of crosslinking; crosslinking conditions (eg, temperature and pH); the molecular weight of the starting HA; and any postcrosslinking modifications—significantly influence the gel's final physicochemical properties, such as its rheology (elasticity, cohesivity, and viscosity). Consequently, these factors can alter the filler's behavior under stress and its tissue-integration profile, which are closely related to its clinical efficacy and potential side effects.^7^ This variability means that even HA fillers with similar active ingredients may yield different clinical outcomes, underscoring the necessity for rigorous comparative studies.

Because the aging population expands and the demand for anti-aging aesthetic treatments continues to rise, there is a clear imperative to broaden the range of available products. The development of new HA fillers, even those sharing similar primary compositions, is essential to foster competition and provide practitioners and patients with a greater choice of safer, potentially more cost-effective, and easier-to-use treatment options, ultimately helping to meet the growing global aesthetic needs.

Pain during injection is a common concern for patients undergoing dermal filler procedures.^8^ This has led to the incorporation of local anesthetics, such as lidocaine, directly into the filler formulation to improve patient comfort. Studies have consistently demonstrated that the addition of 0.3% lidocaine significantly reduces procedural pain and improves patient comfort, without compromising the filler's efficacy or duration of action.^9^

The investigational medical device, SkinPlus-HYAL Implant Lidocaine (BioPlus Co., Ltd, Seongnam-si, Gyeonggi-do, South Korea), is a newly developed crosslinked HA filler designed for the temporary correction of moderate-to-severe NLFs. This study was conducted to demonstrate the noninferiority of the test device compared with the control device (RESTYLANE Lidocaine, a globally recognized HA filler, Dallas, TX) in a head-to-head clinical trial.

## METHODS

### Study Design

This prospective, multicenter, randomized, patient- and evaluator-blinded, paired-design, noninferiority clinical investigation was conducted across 3 centers in the Republic of Korea. The study protocol was preapproved by the Korean Ministry of Food and Drug Safety as a confirmatory stage trial. The IRBs of all participating centers approved the protocol (Seoul National University Hospital, Seoul, South Korea, no. 2211-046-1377, Asan Medical Center Seoul, South Korea, no. S2022-2334-0001, and Soonchunhyang University Bucheon Hospital, Bucheon, South Korea, no. SCHBC-2022-11-009-009). The study was registered under the identification number NCT06305520. Patient recruitment began on March 13, 2023, and the final patient visit was completed on August 7, 2024. The total study period was 48 weeks, comprising a 24-week primary efficacy and safety evaluation period, followed by an additional 24-week long-term safety and efficacy follow-up. Written informed consent for publication of clinical photographs was obtained from all participants.

### Patients

Adult patients aged 19 years or older with bilateral NLFs scoring 3 (moderate) or 4 (severe) on the Wrinkle Severity Rating Scale (WSRS) were eligible for enrollment. Exclusion criteria included a history of bleeding disorders, autoimmune diseases, use of other fillers within 1 year, or use of other wrinkle treatments (including botulinum toxin and chemical peels) within 24 weeks before screening.

### Device Composition and Physicochemical Characteristics

The test device, SkinPlus-HYAL Implant Lidocaine, is a sterile, absorbable dermal filler composed of 2.0% (20 mg/mL) crosslinked HA and 3.2 mg/mL lidocaine hydrochloride. The gel is processed to have a particle size distribution of 500 to 800 μm and is delivered in a 1 mL prefilled COC syringe. The test device employs divinyl sulfone (DVS) as the crosslinking agent. DVS is a bifunctional vinyl compound that forms shorter and stronger crosslink bridges between HA chains.

### Randomization and Blinding

This study was conducted in a patient- and evaluator-blinded manner. Only patients who met all inclusion and exclusion criteria within 2 weeks before device administration were enrolled and assigned a unique patient registration number at baseline (Week 0) by the investigator. The allocation sequence for the test and control devices was determined in advance using a block randomization method generated by an independent statistician not involved in the clinical investigation. Block randomization was performed using the latest version of SAS software to ensure allocation concealment and minimize selection bias. A randomization table with an adequately sized sequence of randomization numbers, accounting for the predetermined block size, was prepared accordingly.

Randomization envelopes were produced based on this table and sequentially numbered. These envelopes were provided to the investigator, who opened them in the order of patient enrollment to determine the allocation of the test and control devices. To maintain blinding, both the patients and the independent evaluators were kept unaware of which product (test or control) was administered to each NLF (left or right) until the end of the study. Three independent, blinded dermatologists assessed the primary efficacy endpoint using only standardized pre- and posttreatment photographs. The investigator, although not blinded, became aware of the allocation by opening the randomization envelope immediately before device administration.

### Interventions

A total of 100 patients were randomized. Using a block randomization schedule generated by an independent statistician, each patient received the test device (SkinPlus-HYAL Implant Lidocaine) in 1 NLF and the control device (RESTYLANE Lidocaine) in the contralateral NLF. The injection technique was standardized across all cases using the linear threading method, performed either in a superior-to-inferior or inferior-to-superior direction. The filler was administered in multiple passes using either a needle or a cannula and was injected into the plane between the subcutaneous fat tissue and the dermis. A total of 1 mL was injected into each NLF.

### Assessments

The efficacy analysis was primarily performed on the full analysis set. Primary efficacy endpoint is the mean difference in the independent evaluator-assessed WSRS score between the test and control groups at Week 24 postprocedure. The WSRS is a validated 5-point photonumeric scale used for the objective assessment of NLF severity.^10^ Secondary efficacy endpoints are included: mean differences in WSRS scores (independent evaluator and investigator assessed) and Global Aesthetic Improvement Scale (GAIS) scores (investigator and patient assessed) at Weeks 8, 16, 24, and 48. GAIS is a validated 5-point scale ranging from “worse” to “very much improved” used for assessing aesthetic changes. At each follow-up visit (Weeks 8, 16, 24, and 48), the investigator and patient were shown patient's standardized baseline (Week 0) photographs and asked to compare them with patient's current appearance in order to independently assess their satisfaction using the GAIS. GAIS scores were recorded separately for each NLF (left and right). Differences in patient-rated Pain Visual Analog Scale (VAS) scores were also collected immediately after, 15 min, and 30 min postinjection.

Safety assessments were performed on the safety set (*n* = 100). Safety endpoints included the incidence and severity of all adverse events (AEs) and adverse device effects, including local AEs at the injection site (eg, bruising, erythema, swelling, pain, tenderness, and itching) monitored throughout the study period.

### Statistical Analysis

The primary hypothesis was tested for noninferiority, setting the margin at 0.29. Additionally, the 2-sample *t*-test was used to compare the mean WSRS scores between the test and control groups at Week 24. A *P*-value of <.05 was considered statistically significant for secondary endpoints.

## RESULTS

### Study Population and Baseline Characteristics

**Figure 1.**
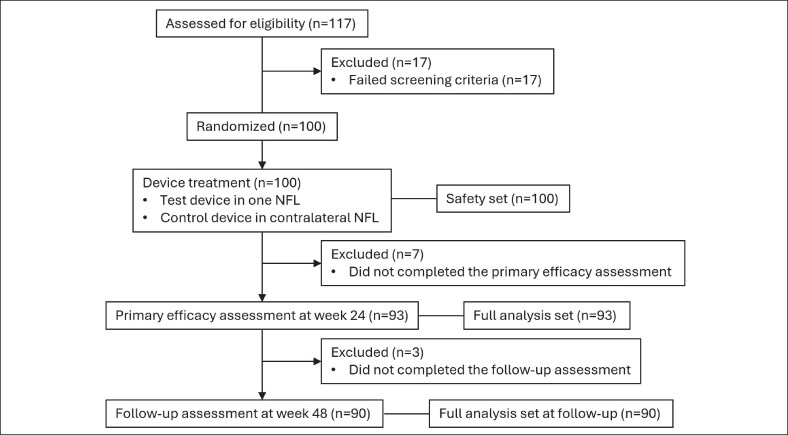
Flow chart of patient disposition.

A total of 117 patients were screened, and 100 were randomized and received treatment, constituting the safety set. The full analysis set for the primary endpoint at Week 24 included 93 patients. The overall study flow, detailing exclusions from the full analysis set, is summarized in Figure 1.

The cohort consisted primarily of females (91%; 91 women and 9 men), with a mean age of 51.04 ± 9.13 years (range, 31.00-72.00 years). The average patient follow-up time was 275.0 ± 131.5 days, with a minimum of 0 days and a maximum of 417 days. Patient demographics are detailed in Table 1.

**Table 1.** Patient Demographics and Baseline Characteristics (*n* = 100)

Characteristic	Statistics	Value
Age (years)	Mean ± SD	51.04 ± 9.13
	Median	50.50
	Range (min, max)	31.00, 72.00
Gender, *n* (%)	Female	91 (91.00)
	Male	9 (9.00)
Height (cm)	Mean ± SD	159.40 ± 7.45
Weight (kg)	Mean ± SD	59.08 ± 9.58

SD, standard deviation.

### Efficacy Results

#### Primary Endpoint (WSRS at Week 24)

**Figure 2.**
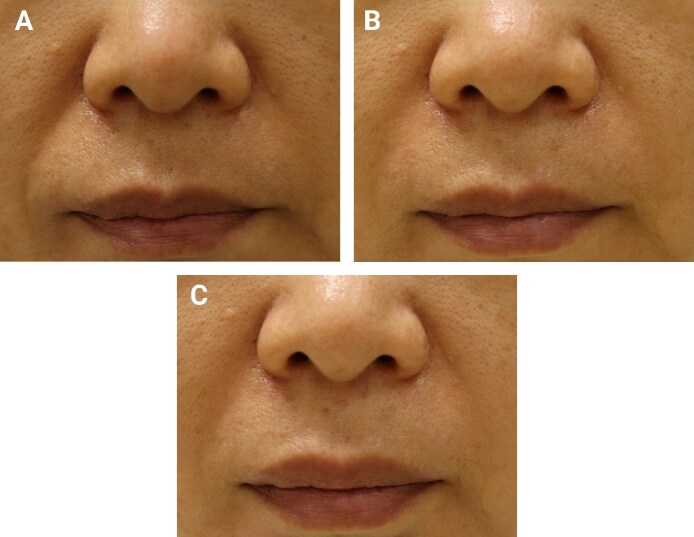
Patient 1 (female, 50 years old). (A) Baseline image. The right nasolabial fold (NLF) was treated with the test device (SkinPlus-HYAL Implant Lidocaine) and the left NLF with the control device (Restylane Lidocaine). (B) Week 24 follow-up and (C) Week 48 follow-up. At all the time points, the Wrinkle Severity Rating Scale scores improved from 3 at baseline to 1 bilaterally at Week 24, with maintenance at 1 through Week 48.

**Figure 3.**
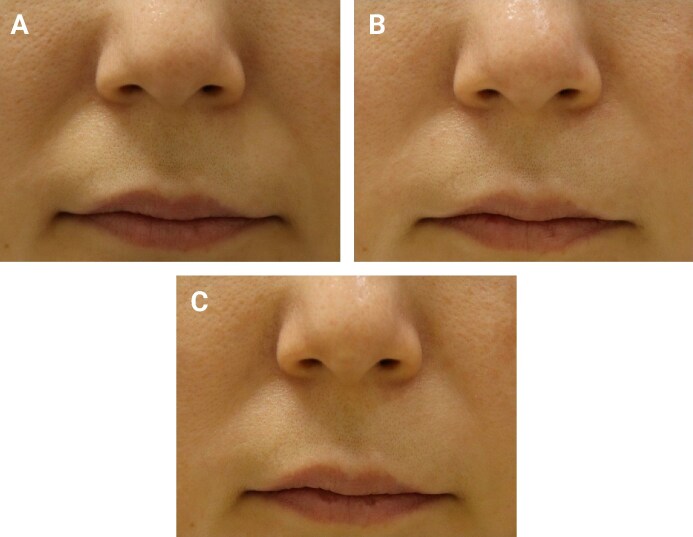
Patient 2 (female, 44 years old). (A) Baseline image. The left nasolabial fold (NLF) was treated with the test device, and the right NLF with the control device. (B) Week 24 follow-up and (C) Week 48 follow-up. Wrinkle Severity Rating Scale scores improved bilaterally from 3 at baseline to 1 at Week 24 and remained stable at 1 at Week 48.

**Figure 4.**
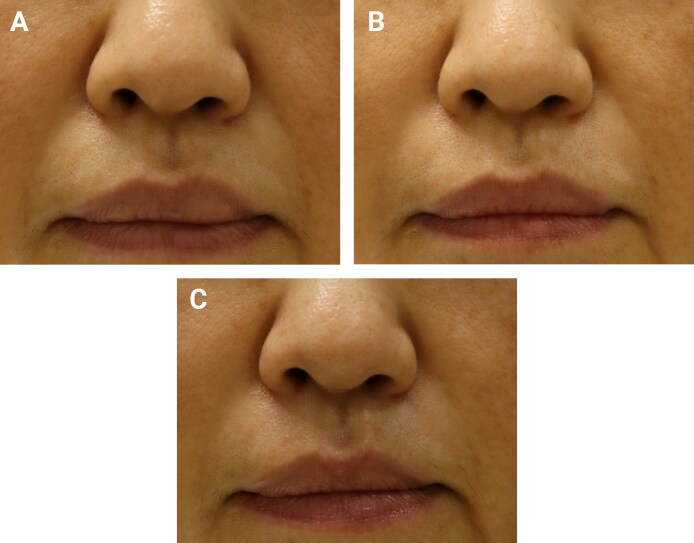
Patient 3 (female, 52 years old). (A) Baseline image. The right nasolabial fold (NLF) was treated with the test device, and the left NLF with the control device. (B) Week 24 follow-up. (C) Week 48 follow-up. Wrinkle Severity Rating Scale scores improved bilaterally from 3 at baseline to 1 at Week 24 and remained at 1 through Week 48.

In the full analysis set, the mean WSRS score at Week 24 postprocedure was 1.85 ± 0.72 for the test device and 1.84 ± 0.68 for the control device. The mean difference (test − control) was 0.01 ± 0.48. The prespecified noninferiority margin was 0.29 WSRS units, derived from previous NLF filler studies and representing a fraction of a clinically meaningful 1-grade change. The 97.5% 1-sided CI (equivalent to a 95% 2-sided CI) upper bound for test − control was 0.2136, meeting noninferiority at Week 24. These primary efficacy outcomes are summarized in Table 2, and representative clinical photographs showing wrinkle improvement at baseline, Week 24, and Week 48 for 3 patients are provided in Figures 2-4.

**Table 2.** Summary of Efficacy Results (Full Analysis Set)

Endpoint	Time point	Test group mean (SD)	Control group mean (SD)	Mean difference (SD)	*P*-value
WSRS (independent rater)	24 weeks	1.85 ± 0.72	1.84 ± 0.68	0.01 ± 0.48	.9664
	8 weeks	1.85 ± 0.81	1.83 ± 0.75	0.02 ± 0.47	.9556
	16 weeks	1.87 ± 0.73	1.97 ± 0.70	−0.10 ± 0.42	.2919
	48 weeks	2.12 ± 0.80	2.08 ± 0.81	0.04 ± 0.45	.6924
WSRS (investigator)	8 weeks	2.27 ± 0.77	2.46 ± 0.73	−0.19 ± 0.63	.0927
	16 weeks	2.29 ± 0.76	2.41 ± 0.77	−0.12 ± 0.53	.2670
	24 weeks	2.35 ± 0.76	2.48 ± 0.77	−0.13 ± 0.52	.2038
	48 weeks	2.48 ± 0.75	2.53 ± 0.74	−0.06 ± 0.46	.5881
GAIS (investigator)	8 weeks	3.52 ± 0.82	3.28 ± 0.80	0.24 ± 0.65	.0522
	16 weeks	3.53 ± 0.79	3.31 ± 0.77	0.22 ± 0.57	.0583
	24 weeks	3.42 ± 0.77	3.19 ± 0.76	0.23 ± 0.47	.0370*
	48 weeks	3.27 ± 0.73	3.10 ± 0.70	0.17 ± 0.43	.1574
GAIS (patient)	8 weeks	3.48 ± 0.84	3.18 ± 0.83	0.30 ± 0.66	.0133*
	16 weeks	3.30 ± 0.84	3.13 ± 0.90	0.17 ± 0.69	.1634
	24 weeks	3.23 ± 0.91	3.10 ± 0.89	0.13 ± 0.71	.3057
	48 weeks	3.03 ± 0.93	2.99 ± 0.92	0.04 ± 0.67	.6941
Pain VAS (patient)	Immediate	10.76 ± 16.98	9.74 ± 15.04	−1.02 ± 10.94	.2336
	15 min	6.51 ± 12.69	6.27 ± 13.43	−0.24 ± 9.16	.6653
	30 min	4.48 ± 10.96	3.99 ± 12.50	−0.49 ± 8.82	.3486

GAIS, Global Aesthetic Improvement Scale; SD, standard deviation; VAS, Visual Analog Scale; WSRS, Wrinkle Severity Rating Scale.

^*^Statistically significant difference (*P* < .05).

#### Secondary Endpoints

The WSRS scores remained comparable between the 2 groups at all subsequent follow-up visits, with no statistically significant differences observed between the mean WSRS scores as assessed by the independent evaluator or the investigator. Statistically significant superiority favoring the test device was observed in investigator-assessed GAIS at Week 24 (*P* = .0370) and patient-assessed GAIS at Week 8 (*P* = .0133). Pain VAS score assessments showed no statistically significant difference between the 2 products. The results of secondary endpoints are presented in Table 2.

### Safety Results

The overall safety profile was favorable, with no serious AEs (SAEs) reported throughout the 48-week study period (*n* = 100). The overall incidence of systemic AEs was 47.00% (47 patients/92 events). Among these, only 6 events in 5 patients (5.0%) were determined by investigators to be possibly related to the investigational devices. The most frequently reported systemic AEs, based on system organ class (SOC), were musculoskeletal and connective tissue disorders (17 events in 13 patients), including myalgia, intervertebral disc protrusion, and osteoarthritis. Other commonly reported systemic AEs included respiratory, thoracic, and mediastinal disorders (12 events in 10 patients), gastrointestinal disorders (10 events in 9 patients), infections and infestations (9 events in 9 patients), and skin and subcutaneous tissue disorders (7 events in 6 patients). Less frequent events were classified under injury and procedural complications, eye disorders, vascular disorders, metabolism and nutrition disorders, and reproductive system disorders. Device-related systemic AEs were limited to 4 cases of skin and subcutaneous tissue disorders (3 patients), 1 general disorder and administration site condition, and 1 infection. Because of the paired design of the study, it was not possible to attribute these events to either the test or control device specifically. Systemic AEs are summarized in Table 3.

**Table 3.** Summary of Systemic Adverse Events (*n* = 100)

System organ class	*n* (%), events
Musculoskeletal and connective tissue disorders	13 (13.0), 17
Respiratory, thoracic, and mediastinal disorders	10 (10.0), 12
Gastrointestinal disorders	9 (9.00), 10
Infections and infestations	9 (9.00), 9
Injury, poisoning, and procedural complications	5 (5.00), 7
Eye disorders	5 (5.00), 5
Vascular disorders	3 (3.00), 4
Metabolism and nutrition disorders	3 (3.00), 3
Skin and subcutaneous tissue disorders	6 (6.00), 7
Reproductive system and breast disorders	3 (3.00), 3
Nervous system disorders	2 (2.00), 2
Neoplasms benign, malignant, and unspecified (including cysts and polyps)	2 (2.00), 2
Psychiatric disorders	2 (2.00), 2
Blood and lymphatic system disorders	1 (1.00), 1
Cardiac disorders	1 (1.00), 1
Endocrine disorders	1 (1.00), 1
General disorders and administration site conditions	1 (1.00), 1
Hepatobiliary disorders	1 (1.00), 1
Renal and urinary disorders	1 (1.00), 1
Surgical and medical procedures	1 (1.00), 1

Local AEs at the injection site were common for both products but were primarily mild to moderate and transient. The total incidence rate for local AEs was statistically significantly higher in the test group (92.00%) compared with the control group (82.00%; *P* = .0355).

Specific local AEs are detailed in Table 4. In the test group, the most frequent local AEs included tenderness (83.00%, 83 events), injection-site pain (82.00%, 82), swelling (71.00%, 71), erythema (51.00%, 51), bruising (48.00%, 48), and pruritus (40.00%, 40). In the control group, the most common events were injection-site pain (60.00%, 60), tenderness (59.00%, 59), swelling (45.00%, 45), erythema (27.00%, 27), bruising (35.00%, 35), and pruritus (25.00%, 25). The incidence of tenderness, injection-site pain, swelling, and erythema was statistically significantly higher in the test group compared with the control group. Conversely, the incidence of bruising was statistically comparable between the test group (48.00%) and the control group (35.00%; *P* = .0621).

**Table 4.** Incidence of Local Adverse Events (*n* = 100)

Local AE	Test group, *n* (%) (events)	Control group, *n* (%) (events)	*P*-value
Any local AE (incidence rate)	92 (92.00)	82 (82.00)	.0355*
Tenderness	83 (83.00)	59 (59.00)	.0002*
Injection-site pain	82 (82.00)	60 (60.00)	.0006*
Swelling	71 (71.00)	45 (45.00)	.0002*
Erythema	51 (51.00)	27 (27.00)	.0005*
Pruritus	40 (40.00)	25 (25.00)	.0235*
Bruising	48 (48.00)	35 (35.00)	.0621

AE, adverse event.

^*^Statistically significant difference (*P* < .05).

## DISCUSSION

This randomized, patient- and evaluator-blinded, paired clinical study was designed to rigorously compare the efficacy and safety of a newly developed crosslinked HA filler, SkinPlus-HYAL Implant Lidocaine, against a market-established control, RESTYLANE Lidocaine. The paired design minimized confounding variables stemming from individual patient differences, enhancing the internal validity of the head-to-head comparison.

The primary efficacy analysis successfully confirmed the noninferiority of the test device to the control device at the primary endpoint of Week 24, with the 97.5% upper confidence limit (0.2136) falling well below the noninferiority margin (0.29). This finding was sustained through the 48-week follow-up, suggesting comparable mechanical properties and persistence between the 2 products for NLF correction.

The distinction between objective and subjective evaluation remains notable. Despite objective equivalence in WSRS, the subjective perception of the aesthetic outcome favored the test device at specific time points, with statistical superiority observed in investigator-assessed GAIS at Week 24 (*P* = .0370) and patient-assessed GAIS at Week 8 (*P* = .0133). One possible explanation for this finding lies in the physicochemical differences between the 2 fillers. The test device's use of DVS as a crosslinking agent—resulting in shorter, stronger intramolecular bridges—confers a more cohesive and elastic gel structure compared with 1,4-butanediol diglycidyl ether (BDDE)-based fillers like RESTYLANE Lidocaine. Higher cohesiveness and viscoelasticity may enhance the filler's ability to integrate uniformly within the tissue, resist migration, and maintain projection over time. These attributes may contribute to improved tactile feel, enhanced moldability, and better aesthetic contouring, which are elements more likely to be appreciated by both clinicians and patients in subjective evaluations such as GAIS. Although these effects were not directly quantified in the current study, they may offer a plausible mechanistic basis for the early and mid-term aesthetic superiority perceived with the test filler. Further studies incorporating rheological measurements and histological analysis may help validate this hypothesis and better characterize how the material properties of HA fillers translate into clinical outcomes.

The safety comparison provided compelling data regarding local AEs. Although the overall local AE rate was statistically higher for the test device (*P* = .0355), the AEs were generally mild and transient, consistent with the known profile of injectable HA fillers, which includes swelling, redness, pain, and bruising. Importantly, all local AEs resolved spontaneously within 2 weeks without the need for medical intervention. Crucially, AEs related to inflammatory response (tenderness, pain, swelling, and erythema) were significantly more frequent in the test group. In contrast, injection-site bruising—an AE often related to needle trauma or the vasodilatory effect of lidocaine—showed no statistical difference between the groups. This suggests that the increased local inflammatory events in the test group may be related to the filler's specific physicochemical formulation, possibly related to HA fragmentation or residual contaminants from the crosslinking or purification process, rather than the injection procedure itself. Different crosslinking and manufacturing methods yield diverse rheological characteristics and purification levels, which are directly implicated in a filler's longevity, clinical performance, and potential for inflammatory side effects. Regarding systemic AEs, musculoskeletal and connective tissue disorders were reported in 13% of patients. These included myalgia, intervertebral disc protrusion, and osteoarthritis, among others. Additional systemic AEs were observed in fewer than 10% of participants. Importantly, all systemic events were reviewed in detail by the investigators. Based on clinical judgment, only a small number of AEs were considered potentially related to the filler injections: 4 cases (in 3 patients) of skin and subcutaneous tissue disorders, 1 case of general disorders and administration site conditions, and 1 case of infections and infestations. However, because of the paired study design—in which both test and control devices were injected into opposite NLFs of the same patient—it was not possible to determine with certainty whether these AEs were associated with the test or control device. Importantly, the absence of any reported SAEs over the entire 48 weeks establishes an acceptable safety profile for the test device.

This study has several limitations that should be considered. First, all enrolled patients were Korean, and the majority (91%) were female, limiting the generalizability of the findings to broader populations across different ethnicities and genders. Although Fitzpatrick skin type was not evaluated in this study, Koreans are generally classified as Fitzpatrick skin types III to IV.^11^ Previous literature has highlighted potential ethnic differences in the incidence of adverse reactions to HA fillers.^12^ Therefore, future clinical trials involving more ethnically and demographically diverse populations are warranted to further evaluate the efficacy and safety profile of the test device. Second, the follow-up period of 48 weeks may be insufficient to detect delayed-onset AEs. Previous studies have reported that certain HA filler–related complications can manifest >1 year after injection.^13^ Thus, extended long-term follow-up will be essential to comprehensively assess the delayed safety outcomes of the product. Lastly, the test group demonstrated a statistically significantly higher incidence of local inflammatory responses, including pain, tenderness, erythema, and swelling. Although these events were generally mild and transient, further studies are needed to determine whether this trend persists over time. Should such a pattern be consistently observed, additional investigations into the immunological or histopathological mechanisms underlying these reactions would be necessary.

## CONCLUSIONS

SkinPlus-HYAL Implant Lidocaine demonstrated noninferior efficacy to the comparator RESTYLANE Lidocaine for the temporary correction of moderate to severe NLFs, with comparable objective results maintained up to 48 weeks. The test device showed superior patient and investigator satisfaction at specific follow-up visits. Although the overall local AE rate was statistically higher for the test device, its overall safety profile was acceptable, with no reported SAEs. These findings support the use of SkinPlus-HYAL Implant Lidocaine as a safe and effective option for aesthetic treatment of NLFs, contributing positively to the growing demand for diverse, safe, and effective anti-aging treatment modalities. In addition, the availability of HA fillers with differing physicochemical properties may help enable more tailored treatment approaches and improve patient accessibility through cost-effective alternatives.
